# Minor Ailments and Pharmacist Management in Ontario, Canada: Attachment and Primary Care

**DOI:** 10.23889/ijpds.v9i5.2702

**Published:** 2024-09-18

**Authors:** Lisa Dolovich, Paul Nguyen, Yasmin Abdul Aziz, Mina Tadrous, Ernie Avilla, Annalise Mathers, Eliot Frymire, Rick Glazier, Kamila Premji, Liisa Jaakkimainen, Tara Kiran, Lynn Roberts, Michael E Green

**Affiliations:** 1 University of Toronto; 2 Queen's University; 3 ICES; 4 University Of Ottawa; 5 St. Michael's Hospital

Minor ailments (MA) are health conditions that can be managed with minimal prescribed treatment and/or self-care strategies. In Ontario, Canada, pharmacists were given authority to deliver service for thirteen MAs on January 1, 2023, with an additional six MAs on October 1, 2023. This study aims to identify patterns of MA service provision and describe characteristics of recipients of MA services.

A cross-sectional analysis of linked health administrative data, including pharmacist billing for MA services, patient demographics, and physician and hospital billing data, was conducted. Delivery of MA services prescribed in Ontario from January 1 to December 31, 2023 were analyzed with key demographics (age, sex, income, new arrival status, comorbidity), health care utilization (ED, hospitalization) and primary care attachment.

In 2023, 547,673 (3.6%) Ontario residents received at least one MA service, with the top 5 services being urinary tract infections (199,282), conjunctivitis (153,021), herpes labialis (46,659), allergic rhinitis (41,521), and dermatitis (39,365). Compared to all Ontario residents, the MA service recipients were more likely to be people with a higher rate of ED visits (41.7% vs. 32.4%), comorbidities (17.5% vs. 9.7%), higher incomes (highest income quintile, 23.1% vs. 20.1%; lowest income quintile, 16.5% vs. 19.4%), and hospitalizations (23.6% vs. 17.6%), and less likely to be newcomers to Ontario (8.7% vs. 13.1%). They were more likely to be attached to a primary care provider (91.3% vs. 84.7%).

This study provides insight into early users of this program, and sheds light on those with systemic vulnerabilities and equity deserving groups.

